# The EMT-activator *ZEB1* is unrelated to platinum drug resistance in ovarian cancer but is predictive of survival

**DOI:** 10.1007/s13577-022-00744-y

**Published:** 2022-07-06

**Authors:** Sophie Rae, Cathy Spillane, Gordon Blackshields, Stephen F. Madden, Joanne Keenan, Britta Stordal

**Affiliations:** 1 Department of Natural Sciences, Middlesex University London, London, UK; 2 Department of Histopathology, St James’ Hospital and Trinity College Dublin, Dublin, Ireland; 3 Data Science Centre, Royal College of Surgeons in Ireland, Dublin, Ireland; 4 National Institute for Cellular Biotechnology, Dublin City University, Glasnevin, Dublin 9, Ireland

**Keywords:** EMT, ZEB1, Ovarian cancer, Cisplatin, Paclitaxel

## Abstract

The IGROVCDDP cisplatin-resistant ovarian cancer cell line is an unusual model, as it is also cross-resistant to paclitaxel. IGROVCDDP, therefore, models the resistance phenotype of serous ovarian cancer patients who have failed frontline platinum/taxane chemotherapy. IGROVCDDP has also undergone epithelial-mesenchymal transition (EMT). We aim to determine if alterations in EMT-related genes are related to or independent from the drug-resistance phenotypes. EMT gene and protein markers, invasion, motility and morphology were investigated in IGROVCDDP and its parent drug-sensitive cell line IGROV-1. *ZEB1* was investigated by qPCR, Western blotting and siRNA knockdown. *ZEB1* was also investigated in publicly available ovarian cancer gene-expression datasets. IGROVCDDP cells have decreased protein levels of epithelial marker E-cadherin (6.18-fold, *p* = 1.58e−04) and higher levels of mesenchymal markers vimentin (2.47-fold, *p* = 4.43e−03), N-cadherin (4.35-fold, *p* = 4.76e−03) and ZEB1 (3.43-fold, *p* = 0.04). IGROVCDDP have a spindle-like morphology consistent with EMT. Knockdown of *ZEB1* in IGROVCDDP does not lead to cisplatin sensitivity but shows a reversal of EMT-gene signalling and an increase in cell circularity. High *ZEB1* gene expression (HR = 1.31, *n* = 2051, *p* = 1.31e−05) is a marker of poor overall survival in high-grade serous ovarian-cancer patients. In contrast, *ZEB1* is not predictive of overall survival in high-grade serous ovarian-cancer patients known to be treated with platinum chemotherapy. The increased expression of *ZEB1* in IGROVCDDP appears to be independent of the drug-resistance phenotypes. *ZEB1* has the potential to be used as biomarker of overall prognosis in ovarian-cancer patients but not of platinum/taxane chemoresistance.

## Introduction

The majority of ovarian-cancer patients present with advanced disease as ovarian cancer has non-specific symptoms [1]. The 5-year survival of women who present with late-stage disease is 29–35% [2, 3]. Advanced ovarian cancer is treated by debulking surgery followed by 3-weekly platinum/taxane chemotherapy [3, 4]. Recurrence, which is associated with resistance to platinum/taxane chemotherapy, is currently incurable in 75% of ovarian-cancer patients [3].

The IGROVCDDP cisplatin-resistant ovarian-cancer cell line is an unusual model, as it is cross-resistant to paclitaxel [5]. IGROVCDDP, therefore, models the resistance phenotype of ovarian-cancer patients who have failed standard frontline platinum/taxane chemotherapy. The paclitaxel resistance in IGROVCDDP is mediated through overexpression of the ABC transporter P-glycoprotein (ABCB1). The IC50s for paclitaxel in the IGROV-1 parental and IGROVCDDP cells over a 6-day exposure are 0.99 ± 1.13 and 127.92 ± 64.76 µg/mL, respectively, a ~ 129-fold change [5]. The platinum resistance in IGROVCDDP cells is multifactorial and involves changes in drug accumulation, glutathione metabolism and DNA repair. The IC50s for cisplatin in the IGROV-1 and IGROVCDDP cells over a 6-day exposure are 42.0 ± 18.0 and 810 ± 345.01 ng/mL, respectively, a ~ 19-fold change [5].

Epithelial to mesenchymal transition (EMT) normally occurs during embryonic development [6]. In EMT, epithelial cells lose their cell–cell adhesion and acquire mesenchymal features such as motility, invasiveness and increased resistance to apoptosis [6, 7]. Cancer cells undergoing EMT can progress from a non-invasive to an invasive phenotype, there can also be a change in cellular morphology associated with this process [6, 8]. Ovarian cancer cell lines have been previously shown to have undergone EMT in association with the development of drug resistance [6, 7, 9–11]. However, the mechanisms of drug resistance are not typically investigated or referred to in detail in the same study. There is also evidence to suggest that EMT occurs in conjunction with clinical ovarian-cancer progression and metastasis [8, 12, 13].

IGROVCDDP has undergone EMT relative to IGROV-1 parental cells. It is the aim of this study to determine if the alterations in EMT-related genes in IGROVCDDP are also involved in the platinum/taxane drug-resistance phenotype. *ZEB1* was identified as differentially expressed in IGROVCDDP by microarray, and has been previously associated with EMT in ovarian-cancer cells [11, 14]. It is important to determine if these changes are associated with or independent of the drug-resistance phenotype to choose appropriate biomarkers of chemoresistance for use in the clinic.

## Results

### IGROVCDDP cells have undergone EMT

**Fig. 1:**
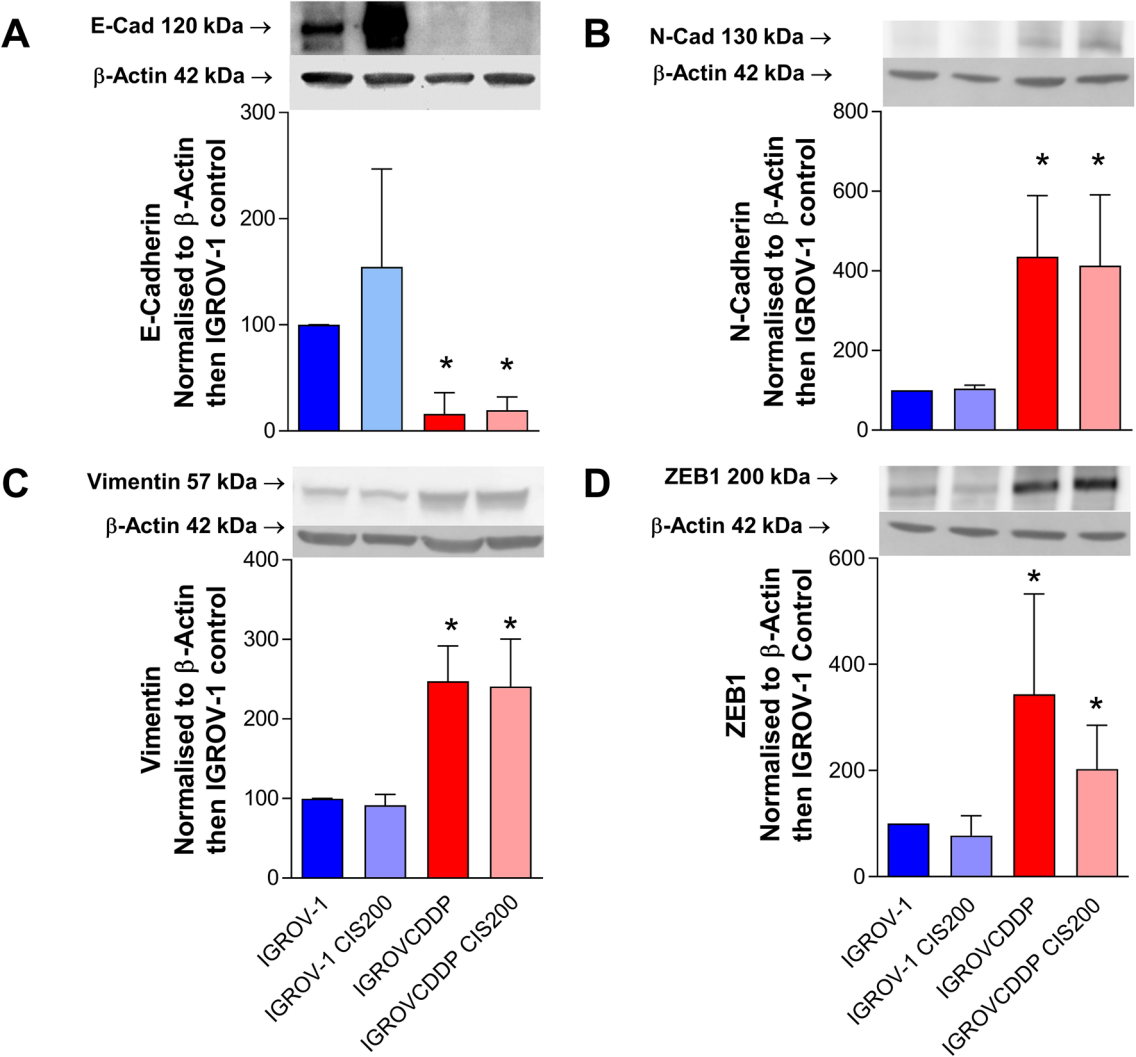
The EMT phenotype in IGROVCDDP cells. Western blots of **A** E-cadherin, **B** N-cadherin, **C** vimentin and **D** ZEB1. IGROV-1 (blue) and IGROVCDDP (red) with and without treatment with 200 ng/mL cisplatin for 72 h (pale blue, pink). Representative images of *n* = 4 biological repeats are shown. Abundance of protein in arbitrary units was normalised to β-actin for each sample and then each biological series was normalised to IGROV-1 control expression. * Indicates significant difference from IGROV-1 *p* < 0.05 Student’s *t* test. No significant difference was observed in IGROVCDDP between the control and the cisplatin-treated cells

IGROV-1 and cisplatin/paclitaxel IGROVCDDP cells were analysed by Affymetrix microarray. The IGROVCDDP cells have mRNA expression changes consistent with EMT relative to parental IGROV-1 cells (Table 1). The gene expression of epithelial marker E-cadherin was decreased and mesenchymal marker N-cadherin was increased (Table 1). IGROVCDDP also has lower protein levels of epithelial marker E-cadherin (6.18-fold, *p* = 1.58e−04, Fig. 1A) and higher levels of mesenchymal markers N-cadherin (4.35-fold, *p* = 4.76e-−3, Fig. 1B) and vimentin (2.47-fold, *p* = 4.43e−03, Fig. 1C) consistent with EMT. Treatment with 200 ng/mL cisplatin did not alter the expression of E-cadherin, N-cadherin or vimentin in the IGROV-1 or IGROVCDDP cell lines (Fig. 1A–C).

**Table 1:** Affymetrix results IGROV-1 vs. IGROVCDDP—differentially expressed EMT-related genes

Gene	Full name	Affymetrix Probe ID	Fold change		FDR
*CDH1*	Cadherin 1 type 1 E-cadherin (epithelial)	7996837	− 7.33	↓↓	1.50E−03
*CDH2*	Cadherin 2 type 1 N-cadherin (neuronal)	8022674	2.14	↑↑	8.69E−04
*VIM*	Vimentin	7926368	− 1.29	↓	0.045
*TWIST1*	Twist homolog 1 (Drosophila)	8138442	− 4.20	↓↓	2.07E−03
*ZEB1*	Zinc finger E-box binding homeobox 1	7926916	1.37	↑	9.25E−03
*SIP1 (ZEB2)*	Survival of motor neuron protein interacting protein 1	7974054	1.40	↑	1.56E−03

↑↑—increased expression, ↓↓—decreased expression, ↑—significant increase less than 2.0-fold, ↓—significant decrease less than 2.0-fold

The mRNA expression of several transcription factors associated with EMT regulation were also altered in IGROVCDDP cells. The mRNA expression of *TWIST1* was significantly decreased, while *ZEB1* and *SIP1(ZEB2)* gene expression were significantly increased (Table 1). The protein expression level of ZEB1 was also significantly increased in IGROVCDDP (3.43-fold, *p* = 0.04, Fig. 1D). Treatment with a low-dose of cisplatin for 72 h did not significantly alter the protein expression of ZEB1 in the IGROV-1 or IGROVCDDP cells (Fig. 1D).

**Fig. 2:**
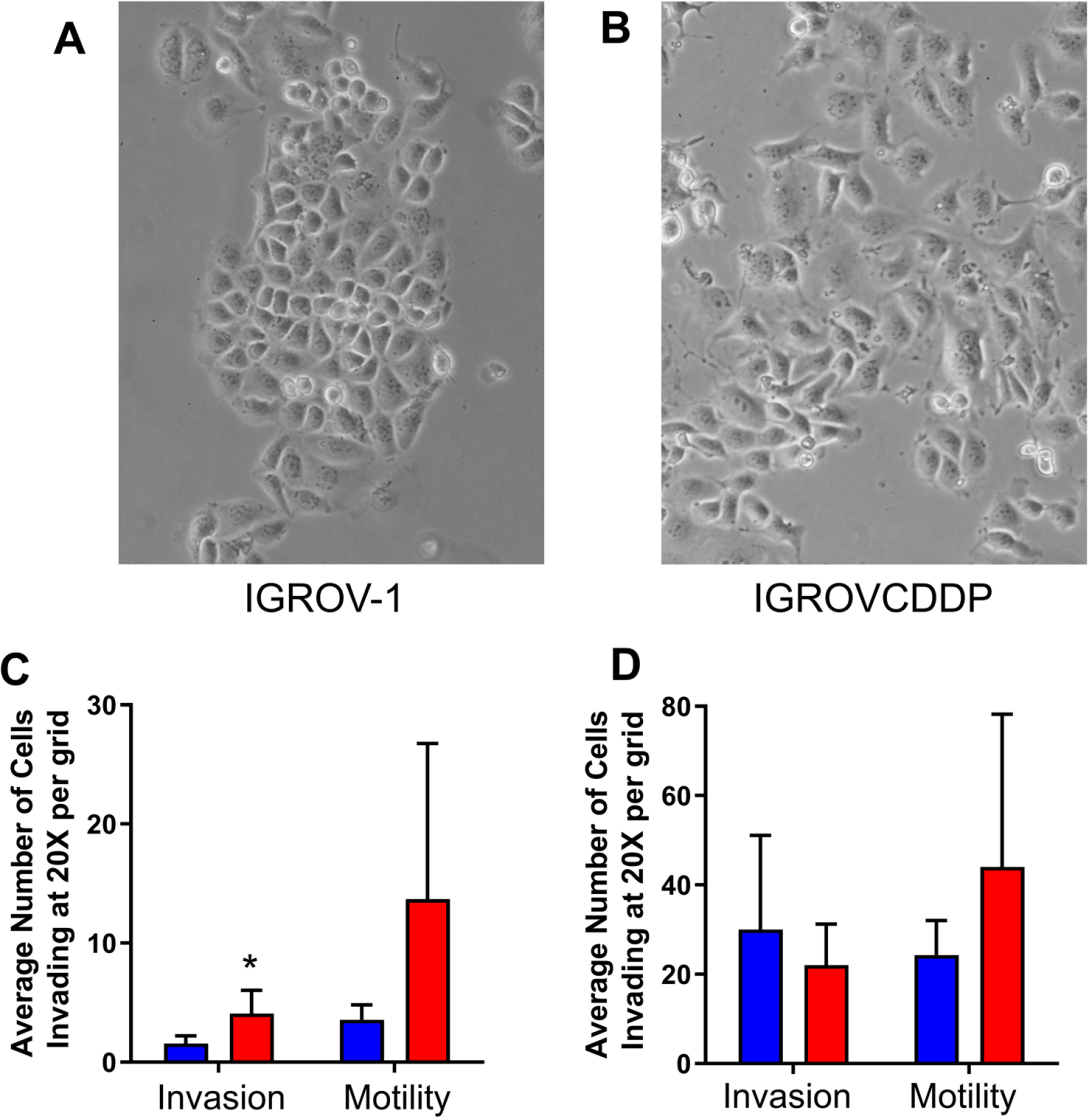
Cellular morphology and invasion. Morphology images of **A** IGROV-1 and **B** IGROVCDDP. Images were taken of 70% confluent cells with ×100 magnification. Invasion and motility in IGROV-1 (blue) and IGROVCDDP (red) cells at C) 24 h and D) 48 h. Average of *n* = 6 biological repeats is shown. * Indicates significant difference from IGROV-1 *p* < 0.05 Student’s *t* test

The change in cell morphology of IGROVCDDP from IGROV-1 is also consistent with EMT (Fig. 2A and B). In parental IGROV-1 cells, the shape of marginal cells was rounded, showing little formation of pseudopodia. In contrast, the morphological changes observed in IGROVCDDP cells include increased loss of cell polarity causing a spindle-shaped morphology in some cells and increased formation of pseudopodia.

The IGROVCDDP cells are more invasive than IGROV-1 at 24 h (2.61-fold, *p* = 0.008, Fig. 2C). Comparison between invasion and motility shows that for both the IGROV-1 and IGROVCDDP cells there is an increase in motility over invasion suggesting the cells are restricted in invasion by their ability to break down the Matrigel extracellular matrix (Fig. 2C). The motility for IGROVCDDP tended to be higher than IGROV-1 but this was not significant (Fig. 2C). At 48 h the IGROV-1 cells tend to be more invasive than the IGROVCDDP cells, but the data were more variable and the difference was not significant (Fig. 2D).

### ZEB1 knockdown in IGROVCDDP alters the gene expression of EMT markers and cellular morphology but not chemoresistance

**Fig. 3:**
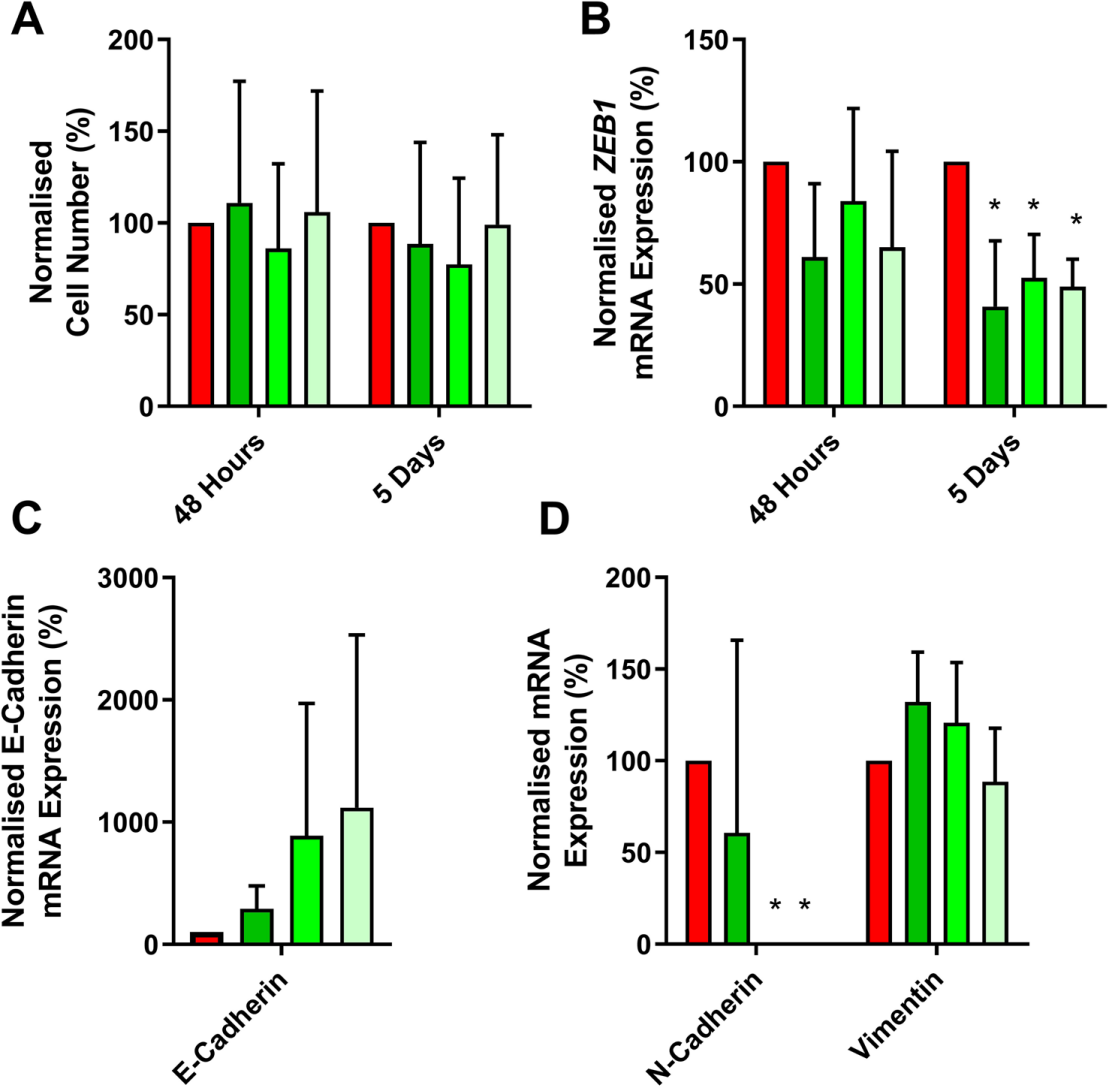
Cell growth and mRNA expression after *ZEB1* siRNA knockdown in IGROVCDDP cells. **A** Cell growth in IGROVCDDP at 48 h and 5 days in response to siRNA knockdown. **B** *ZEB1* gene expression in response to *ZEB1* siRNA knockdown at 48 h and 5 days. **C** E-cadherin; **D** N-cadherin and vimentin gene expression in response to *ZEB1* siRNA knockdown at 5 days. Scramble control (red) with three different *ZEB1* siRNAs (shades of green). * Indicates a significant difference from IGROVCDDP scramble control *p* < 0.05 Student’s *t* test

We investigated the effect of siRNA knockdown of *ZEB1* in IGROVCDDP. *ZEB1* knockdown was examined by qPCR at 48 h and 5 days post-treatment with siRNAs. Cell viability was good post-siRNA transfection and there was no change in cell growth compared to the scramble control at 48 h or 5 days (Fig. 3A). *ZEB1* gene expression tended to be reduced at 48 h post-siRNA transfection (61–84%) but this was not significant (Fig. 3B). *ZEB1* gene expression was significantly knocked down at 5 days post-siRNA transfection for all 3 siRNAs (41–52%).

The gene expression of epithelial marker E-cadherin and mesenchymal markers vimentin and N-cadherin were also examined in the 5-day *ZEB1* siRNA knockdown samples by qPCR. e-Cadherin expression tended to increase in response to knockdown but this was not significant due to high variability (Fig. 3C). There was no change in vimentin in response to knockdown (Fig. 3D). In contrast, the expression of N-cadherin was significantly decreased to a non-detectable level in 2 out of the 3 *ZEB1* siRNAs (Fig. 3D).

**Fig. 4:**
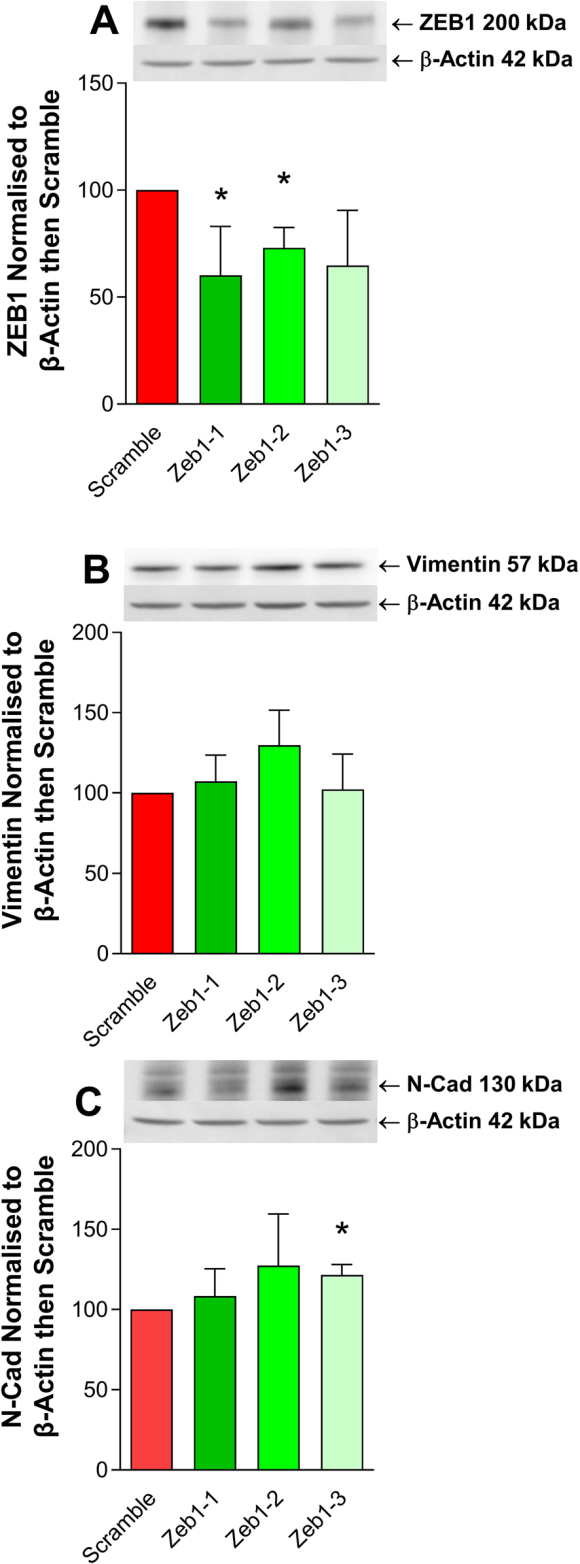
Protein expression after ZEB1 siRNA knockdown in IGROVCDDP. **A** ZEB1, **B** vimentin and **C** N-cadherin protein expression in response to *ZEB1* siRNA knockdown at 3 days. IGROVCDDP scramble control (red) and three different ZEB1 siRNAs (shades of green). Representative images of *n *= 4 biological repeats are shown. Abundance of protein in arbitrary units was normalised to β-actin for each sample and then each biological series was normalised to IGROVCDDP scramble control expression. * Indicates significant difference from IGROVCDDP scramble control *p* < 0.05 Student’s *t* test

The protein expression of ZEB1 was decreased to 60–73% of the scramble control at 72-h post transfection (Fig. 4A). The knockdowns with *ZEB1-1* and *ZEB1-2* siRNA were significant knockdowns (*p* = 0.04 and 8.0e−03, respectively), *ZEB1-3* approached significance (*p* = 0.08). The protein expression of mesenchymal markers vimentin and N-cadherin were examined in the *ZEB1* knockdown samples by Western blot (Fig. 4B and C). There was no change in vimentin. *ZEB1-2* tended to increase N-cadherin and a small but significant increase was observed in response to *ZEB1-3* (1.21-fold, *p* = 0.004, Fig. 4C).

**Fig. 5:**
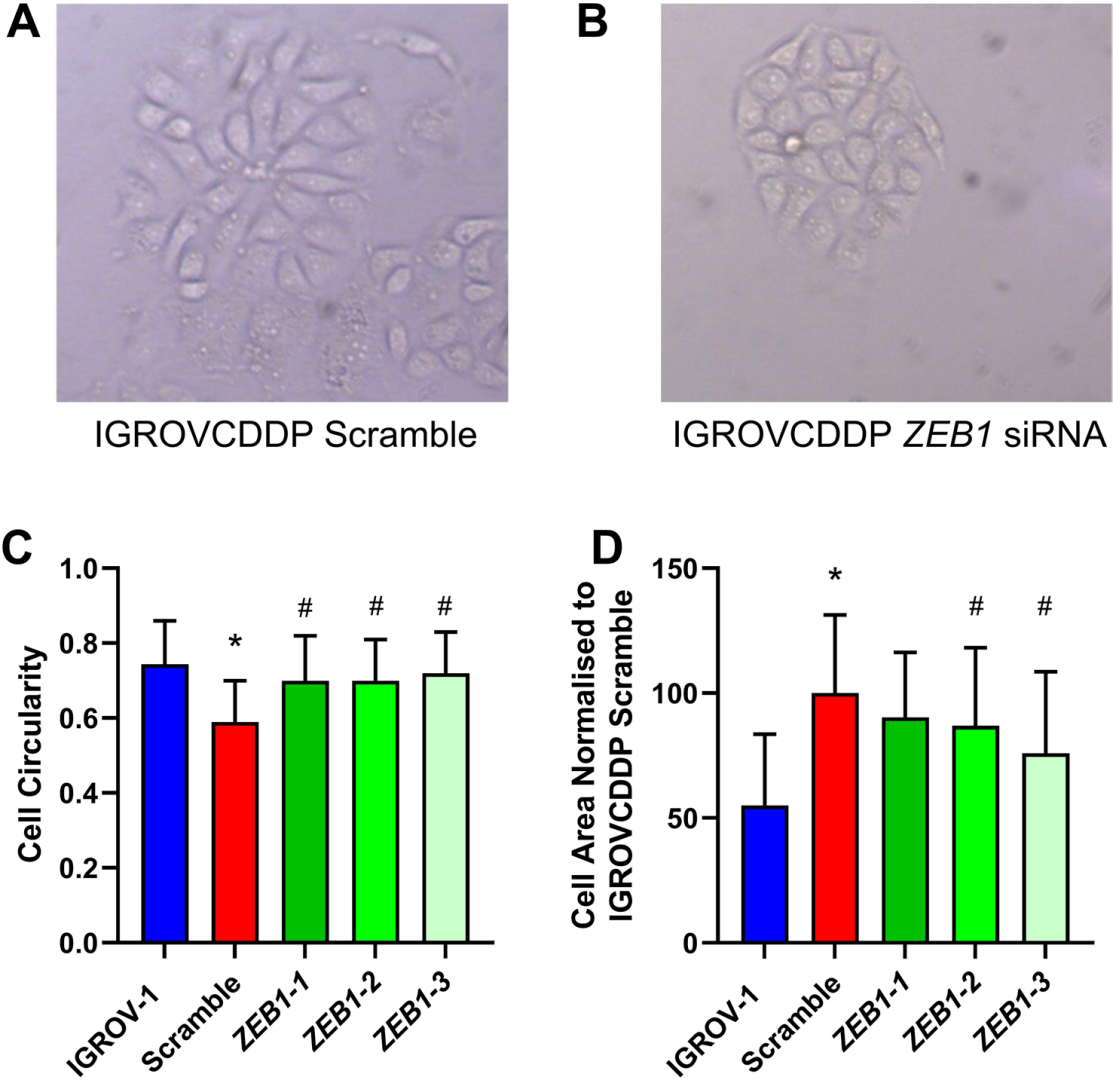
Cellular Morphology after ZEB1 siRNA knockdown in IGROVCDDP. Morphology images of **A** IGROVCDDP Scramble and **B** IGROVCDDP ZEB1 siRNA knockdown, images were taken at 5-days post transfection with ×100 magnification. Image J analysis of **C** Cell circularity and D) Cell Area was performed on images from *n* = 3 biological repeats capturing a minimum of 50 cells per image. IGROV-1 (blue), IGROVCDDP Scramble control (red) with three different *ZEB1* siRNAs (shades of green). * Indicates significant difference from IGROV-1 *p* < 0.05 Student’s *t* test. # Indicates significant difference from IGROVCDDP scramble control *p* < 0.05 Student’s *t* test

The IGROVCDDP cells treated with the siRNA scramble control retain their spindle-like morphology (Fig. 5A). In contrast, the cells treated with the *ZEB1* siRNA have a more epithelial-like morphology with colonies of cells clumped more tightly together suggesting a reversal of the EMT phenotype (Fig. 5B). Morphology images were analysed using Image J [15]. Cell circularity was examined, a figure of 1 indicates a perfect circle. IGROVCDDP cells are less circular than IGROV-1 reflecting their spindle-like morphology (0.59 ± 0.11 vs 0.74 ± 0.11; *p* = 2.74e−09). The *ZEB1* siRNA treatment returns the IGROVCDDP cells to a similar circularity as IGROV-1 (Fig. 5C). IGROVCDDP cells are also larger than IGROV-1 cells, the *ZEB1* siRNA knockdown decreases cell size, but not quite to the size of IGROV-1 (Fig. 5D).

**Fig. 6:**
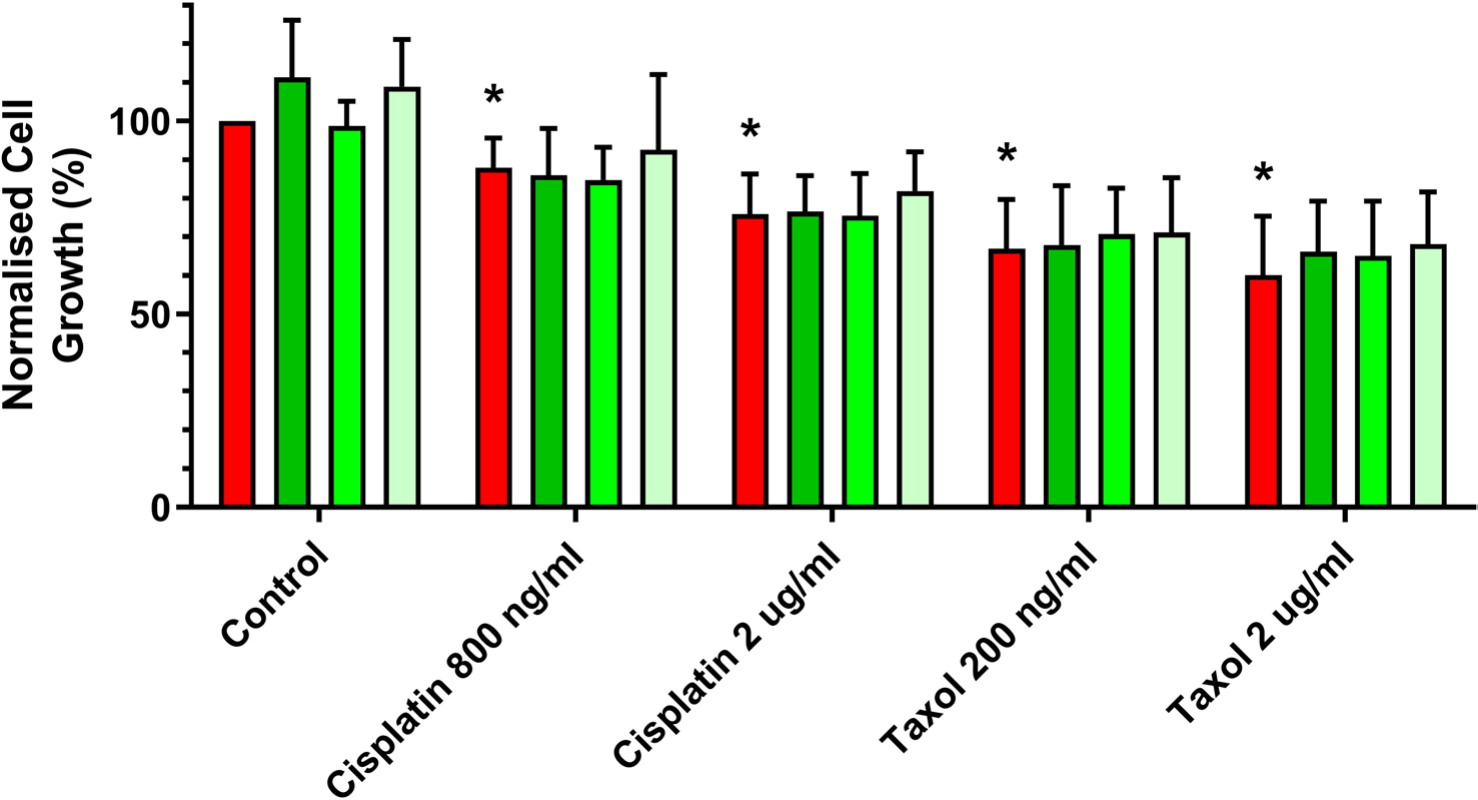
Cytotoxicity after ZEB1 siRNA knockdown in IGROVCDDP. Response to treatment with 800 ng/mL and 2 µg/mL cisplatin as well as 200 ng/mL and 2 µg/mL taxol in ZEB1 siRNA knockdown cells. IGROVCDDP Scramble control (red) with three different *ZEB1* siRNAs (shades of green). * Indicates a significant drop in cell viability compared to IGROVCDDP scramble treatment control *p* < 0.05 Student’s *t* test

The response of IGROVCDDP to cisplatin (800 ng/mL, 2 µg/mL) or taxol (200 ng/mL, 2 µg/mL) treatment over 5 days was not significantly altered by *ZEB1* siRNA knockdown (Fig. 6). Drug treatment induced a significant drop in cell viability but the *ZEB1* siRNA treated cells responded in the same way as the scramble controls (Fig. 6).

### ZEB1 is predictive of overall survival but not in ovarian-cancer patients treated with platinum.

**Fig. 7:**
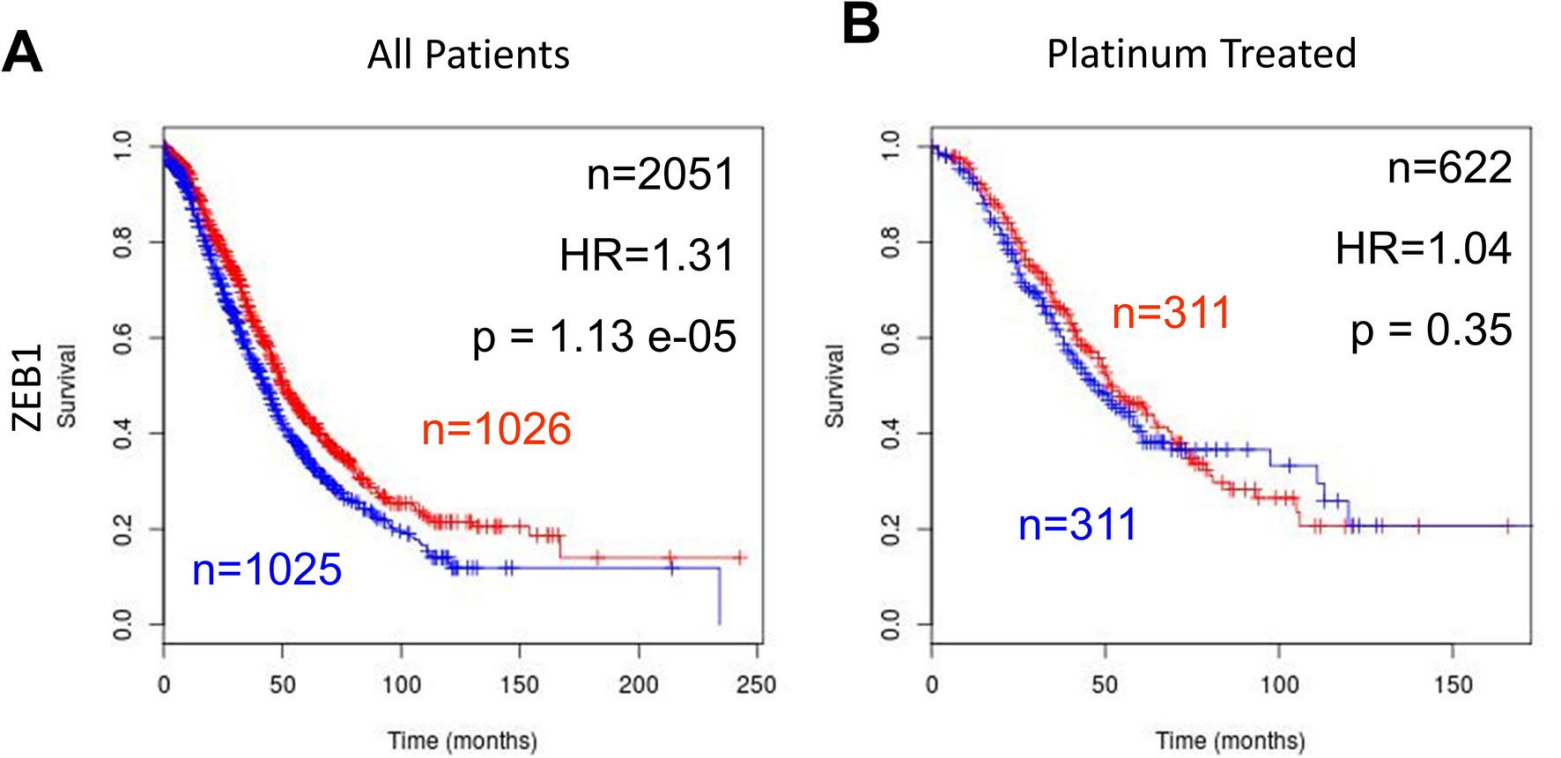
Prognostic role of *ZEB1* gene expression in high-grade serous ovarian cancer. **A** *ZEB1*—high expression (blue) is associated with poor overall in ovarian cancer (HR = 1.31, n = 2051, *p* = 1.13e−05). **B** *ZEB1*—high expression (blue) is not prognostic of overall survival in platinum-treated ovarian-cancer patients

*ZEB1* was analysed in a meta-survival analysis of publicly available gene expression data from high-grade serous ovarian-cancer patients. Median expression of the gene in question was used to dichotomise the data and overall survival was chosen as the survival end point. A hazard ratio of greater than 1 indicates a negative effect on survival and a hazard ratio of less than one has a positive effect. The higher the hazard ratio the greater the effect the gene has on survival. *ZEB1* was individually predictive of overall survival in ovarian cancer. (HR = 1.31, *n* = 2051, *p* = 1.13e−05, Fig. 6A). In contrast, ZEB1 was not predictive of overall survival when the dataset of ovarian-cancer patients was limited to those confirmed to be treated with platinum chemotherapy ((HR = 1.04, *n* = 622, *p* = 0.35, Fig. 6B). Similarly, *ZEB1* was not predictive of overall survival in taxane treated patients (HR = 0.94, *n* = 516, *p* = 0.66) or in patients treated with both platinum and taxane (HR = 0.94, *n* = 515, *p* = 0.67). *ZEB1* was also investigated with progression-free survival as an end point and was not found to be individually prognostic in high-grade serous ovarian-cancer patients (Fig. 7).

## Discussion

We have demonstrated that the IGROVCDDP cisplatin/paclitaxel-resistant cell line has undergone EMT relative to parental IGROV-1 cells. The epithelial marker E-cadherin is decreased and mesenchymal markers N-cadherin is increased at both the gene and protein level (Table 1, Fig. 1A, B). Morphological changes (Fig. 2A and B) are also consistent with a shift to a mesenchymal phenotype. IGROVCDDP cells are, therefore, similar to other chemoresistant ovarian-cancer cell lines which have undergone EMT [6, 7, 9, 10]. E-cadherin is not expressed in normal ovarian surface epithelium. Early-stage epithelial ovarian-cancer is associated with a gain of epithelial features including E-cadherin expression; tumour progression is associated with a reacquisition of mesenchymal features [13]. IGROV-1 cells express E-cadherin. The loss of E-cadherin expression in IGROVCDDP and gaining of other mesenchymal markers suggests that IGROVCDDP cells model a progressed form of ovarian cancer, including drug resistance.

In the IGROVCDDP cells there was no change in the mRNA expression of transcription factors associated with EMT Snail and Slug (data not shown) and TWIST1 is decreased (Table 1). This is in contrast to other ovarian-cancer models of chemoresistance which usually show increases in Snail, Slug and TWIST in association with their EMT phenotypes [6, 7, 9, 10]. This suggests that *ZEB1* is the primary transcription factor driving EMT in the IGROVCDDP cells.

Interestingly, low-level cisplatin treatment of IGROV-1 did not modulate the expression of EMT markers; E-cadherin expression tended to increase with cisplatin treatment rather than decrease (Fig. 1A), and there was no change in vimentin or N-cadherin expression (Fig. 1B, C) The IGROVCDDP cells were induced into EMT through repeated cisplatin treatment over many months of cell culture [16], they are stably drug resistant and are grown in the absence of cisplatin. The mRNA and protein changes associated with EMT are, therefore, maintained in IGROVCDDP in the absence of the EMT-inducer cisplatin. Short-term low-dose cisplatin (200 ng/mL), similar to that used in the original development of the IGROVCDDP resistant cell line, is not sufficient to induce an EMT-like state in IGROV-1 parental cells. The dose of 200 ng/mL cisplatin is clinically relevant [17, 18] and represents an IC_25_ in IGROV-1 and IC_70_ in IGROVCDDP. This dose has also been optimised to show the greatest difference between cell lines while still yielding enough cells in the sensitive IGROV-1 cell line.

### ZEB1, platinum and taxane resistance and EMT

ZEB1 is a transcription factor that can regulate the suppression of E-cadherin and induce EMT [19]. The increase in ZEB1 protein expression (Fig. 1D) could, therefore, be one of the factors mediating EMT in IGROVCDDP. Knockdown of *ZEB1* by siRNA does not impact chemoresistance or alter the growth rate of cells (Fig. 3A, E). In contrast, *ZEB1* siRNA knockdown decreases the gene expression of mesenchymal marker N-cadherin (Fig. 3D) and causes a shift to epithelial-like morphology (Fig. 5). This suggests that *ZEB1* is one of the drivers of the EMT phenotype in IGROVCDDP, but that *ZEB1* is not involved in the mechanism of cisplatin or paclitaxel resistance in IGROVCDDP (Fig. 3E).

*ZEB1* knockdown has been shown to reverse cisplatin resistance in SKOV3/CDDP ovarian carcinoma [20] and SGC7901/DDP gastric carcinoma cells [21]. The mechanisms of cisplatin resistance in SKOV3/CDDP has been linked to the expression of SLC3A2, a type 2 transmembrane cell surface molecule [20]. The mechanisms of cisplatin resistance of SGC7901/DDP include upregulation of the PI3K/Akt pathway and decreased apoptosis [22]. In contrast, the known mechanisms of platinum resistance in IGROVCDDP, include increased expression of glutathione-related genes, copper transporters and BRCA1 [5]. Therefore, ZEB1’s role in cisplatin resistance may be very mechanism specific. It is unclear if SGC7901/DDP or SKOV3/CDDP cells are cross-resistant to paclitaxel like IGROVCDDP cells. Paclitaxel has been studied in SGC7901/DPP cells but its relative resistance to parental SG7901 cells was not reported [23, 24]. Paclitaxel was not examined in the SKOV3/CDDP cells when they were developed [25].

*ZEB1* gene knockdown has been shown to sensitise ovarian cell lines ES-2, SVOV3 and NOS3TR to paclitaxel [26]. The ES-2 and SKOV3 cell lines were relatively drug-sensitive models of intrinsic drug resistance in ovarian cancer, whereas the NOS3TR cells are an acquired model of resistance. It is unclear what mechanism of paclitaxel resistance is being altered in all three models [26]. Increased protein expression of ZEB1 was observed in two paclitaxel-resistant ovarian-cancer cell lines OV3R-PTX and SK3R-PTX [11]. *ZEB1* was knocked down in these cell lines as part of a large study into the EMT mechanism but response to chemotherapy treatment was not investigated [11]. The known mechanisms of taxane resistance in IGROVCDDP, is increased expression of P-glycoprotein [5]. Therefore, *ZEB1*’s role in paclitaxel resistance may be very mechanism specific. It is unclear if NOS3TR, OV3R-PTX or SK3R-PTX cells are cross-resistant to cisplatin like IGROVCDDP cells as no studies could be found examining cisplatin resistance in these models.

A study on ZEB1 responsive genes in a panel of 38 lung-cancer cell lines found high levels of correlation with EMT-associated genes [27], but no correlation with genes that contribute to the mechanisms of resistance in IGROVCDDP such as P-glycoprotein, glutathione-related genes, copper transporters and BRCA1 [5]. This suggests that the transcription factor ZEB1 does not have a consistently strong influence on platinum and taxane drug-resistance pathways and is consistent with our findings that *ZEB1* knockdown does not alter platinum and taxane cross resistance.

### ZEB1 is predictive of overall survival in ovarian cancer, but not in platinum-treated patients.

The IGROV-1 cell line was obtained from a 47-year-old woman who had stage III ovarian cancer [28]. The histological profile was described as with multiple differentiations, primarily endometrioid with some serous clear cells and undifferentiated foci [28]. This histological profile would normally be suggestive of Type I ovarian cancer [29]. However, the BRCA1 and p53 mutations suggests that it is a Type II high-grade serous carcinoma of the SET (Solid, pseudo-endometrioid and transitional cell carcinoma-like morphology) subtype [29–31]. Our meta-survival analysis of publicly available ovarian cancer was performed on data from 2051 samples across 12 datasets [32–43]. Histological information was available on all of the datasets (Table 2). High-grade serous ovarian cancer was selected for in most studies, if it was not it was the dominant subtype. Therefore, this dataset was an appropriate clinical comparison for the IGROV-1 and IGROVCDDP cells.

**Table 2:** Meta-survival analysis of publicly available ovarian-cancer datasets

GEO Accession	Refs.	Sample number	Serous ovarian cancer %	Individual patient data available							
				Age	Residual tumour information	Chemotherapy treatment information	FIGO stage	Histology	Tumour grade	Survival	
										PFS	OS
GSE13876	[34]	157	100	✔	✖	✖	✖	✔	✖	✖	✔
GSE14764	[35]	80	83.8	✖	✔	✖	✔	✔	✔	✖	✔
GSE17260	[37]	110	100	✖	✖	✖	✖	✔	✔	✔	✔
GSE18520	[36]	53	100	✖	✖	✖	✖	✖	✖	✖	✔
GSE9899	[42]	285	92.6	✔	✔	✔	✔	✔	✔	✔	✔
GSE19161	[39]	61	98	✖	✖	✔	✖	✖	✖	✖	✔
GSE19829	[43]	70	92.9	✖	✖	✖	✖	✖	✖	✖	✔
GSE26712	[33]	185	100	✖	✖	✖	✖	✖	✖	✖	✔
GSE30161	[40]	58	85	✔	✖	✔	✖	✔	✖	✔	✔
GSE31245	[32]	57	92	✖	✖	✖	✖	✖	✖	✖	✔
GSE32062	[38]	260	100	✖	✖	✔	✖	✔	✔	✔	✔
TCGA	[59]	562	100	✖	✖	✔	✖	✖	✖	✖	✔

*PFS* progression-free survival, *OS* overall survival, *TCGA* The Cancer Genome Atlas

High gene expression levels of *ZEB1* are predictive of poor overall survival in high-grade serous ovarian-cancer patients (Fig. 4A). This is consistent with other studies which show that high protein levels of ZEB1 are associated with poor progression-free [44] and overall survival in ovarian cancer [26]. The Li et al. study also showed that ZEB1 was highly associated with FIGO stage, the more advanced the metastases the higher the ZEB1 protein expression [44]. In contrast, in this study we found that *ZEB1* gene expression is not predictive of survival in high-grade serous ovarian-cancer patients confirmed to be treated with platinum chemotherapy (Fig. 6B). An analysis of chemotherapy treatment was not performed in Li et al.[44] or Sakata et al. [26]. This clinical finding is consistent with our data in IGROVCDDP. ZEB1 may be an important component of maintaining the more aggressive mesenchymal phenotype of IGROVCDDP but not drug resistance.

Ovarian cancer patients whose cancers grow while receiving platinum-based chemotherapy are considered very platinum resistant [45]. Whereas ovarian cancers that recur 6 or more months after platinum treatment are considered platinum sensitive and platinum-based chemotherapy can be given again [46]. There is uncertainly if ovarian cancer recurring within 6 months of platinum treatment is truly platinum resistant or are clinically aggressive independent of platinum resistance. Case studies have shown women who have responded well to platinum retreatment despite relapsing within 6 months of platinum treatment [45]. It is our hope that markers like *ZEB1* could be useful in determining which ovarian cancers are likely to respond to platinum retreatment*.*

## Conclusions

ZEB1 may be a useful as marker of progression, invasion and metastasis in ovarian cancer which is associated with poor overall survival. ZEB1 expression may occur concurrently with chemoresistance in ovarian cancer but is not a marker of the platinum/taxane cross resistance phenotype. ZEB1 may be useful as a marker for platinum retreatment in recurrent ovarian cancer.

## Materials and methods

### Cell culture and cytotoxicity assays

The human IGROV-1 ovarian-cancer cell line and its cisplatin-resistant variant IGROVCDDP were obtained from Prof. Jan Schellens, of the Netherlands Cancer Institute [16, 47]. IGROV-1 and IGROVCDDP cells were grown in antibiotic and chemotherapy-free RPMI (Sigma) with 10% FCS (Lonza). All cell lines were maintained in a humidified atmosphere with 5% CO_2_ at 37 °C. All cultures were tested routinely and were mycoplasma-free [48]. STR fingerprinting was used to confirm the identity of the cell lines. Cytotoxicity assays were performed as previously described [5]. Cisplatin was obtained from the St. James’ Hospital Pharmacy; Taxol was obtained from Sigma.

### Affymetrix arrays

Cells (1.25 × 10^6^ cells/10 cm dish) were plated and allowed to attach and grow for 3 days to reach 70–80% confluence. The cells were then trypsinised, washed in 10 mL PBS, centrifuged and the supernatant removed. The cell pellets were stored at − 80 °C prior to analysis. Total RNA was prepared using the RNeasy Mini Kit (Qiagen, UK). Affymetrix arrays were performed on biological triplicate samples of IGROV-1 and IGROVCDDP cells. A total of 400 ng of total RNA was reverse transcribed, fragmented and biotin labelled following recommended Affymetrix protocols. All samples run on the arrays had an RNA Integrity Number (RIN) > 9.5 (Bioanalyzer, Agilent), indicating that the RNA was of high quality. Samples were prepared according to the manufacturer’s instructions. Quality control metrics were carried out based on the Affymetrix quality control white paper [49]. Single stranded fragmented, biotin labelled DNA was hybridised to GeneChip^®^ Human Gene 1.0 ST Arrays (Affymetrix). Hybridised arrays were scanned on an Affymetrix GeneChip^®^ Scanner 3000 7G (Affymetrix).

Analysis and comparison of Affymetrix array data was performed using Bioconductor software libraries (www.bioconductor.org). The oligo package [50] was used to import data from CEL files and compute RNA expression values [50–52]. Differential expression analysis of the RNA expression values was performed using RankProd [53], a non-parametric statistical method for identifying significantly de-regulated genes based on the estimated percentage of false predictions. The RankProd method has been shown to perform well in cases where datasets had low numbers of samples or high levels of noise [54]. De-regulated genes were identified as those with a log-based fold-change in expression value of 1.0 or more, using a significance threshold *p* value of 0.05, adjusted for multiple testing. Gene annotation was provided by annaffy [55].

### Invasion and motility assays

A modified version of the Boyden chamber invasion assay was performed [56]. A cell suspension of 1 × 10^5^ cells/mL was incubated on Matrigel (1 mg/mL) pre-coated inserts at 37 °C for 24 h. The Matrigel and cells that had not migrated were removed. The inserts containing migrated cells, were stained with 0.25% crystal violet for 10 min, rinsed and dried. Invasive potential was assessed by counting the stained cells on the inserts. Motility was measured with a Boyden chamber as above but without the addition of Matrigel.

### siRNA knockdown—reverse transfection

ZEB1 siRNAs (Table 3, Applied Biosystems) or a scramble control (Applied Biosystems #4611) were prepared to a final concentration of 30 nM in Opti-MEM Reduced-Serum Media (Life Technologies). The volume of 30 nM siRNA used was appropriate for the size of well (Table 4). An equal volume of Lipofectamine was added to the plate (Table 4) and the plate was gently rocked followed by a 15-min incubation to allow transfection complexes to form. A cell suspension of IGROVCDDP at a density of 2 × 10^4^/mL was then added and plates were incubated at 37 °C with 5% to allow cells to attach overnight. The next day the media was changed an either replaced with drug-free complete RPMI or complete RPMI with cisplatin or taxol. Following 5 days of incubation cells in 96-well plates were analysed by cytotoxicity assay [5]; cells in 6-well plates were trypsinised, washed in 1 mL PBS, transferred to a sterile Eppendorf tube and frozen for RNA extraction or Western Blot Analysis at various time points.

**Table 3:** Sequences of ZEB1 siRNA molecules

SiRNA	Cat no.	Lot no.	ID	Sense (5′–3′)	Antisense (5′–3′)
Zeb1-1	4392420	AS0219/1	S229972	GGACAGCACAGUAAAUCUtt	UAGAUUUACUGUGCUGUCCtg
Zeb1-2	4392420	AS0219/2	S229970	GGAAGAACGUGACAGCACtt	UGUGCUGUCACGUUCUUCCgc
Zeb1-3	4392420	AS0219/0	S229971	GGUAGAUGGUAAUGUAAUAtt	UAUUACAUUACCAUCUACCgc

**Table 4:** Reverse transfection conditions

Size of well	30 nM siRNA	Lipofectamine	IGROVCDDP cells (2 × 10^4^/mL)
96-well	25 µL	25 µL	100 µL
6-well	250 µL	250 µL	2.5 mL

### qPCR

Frozen cell pellets from siRNA knockdown were re-suspended in 200 µL PBS and total RNA was extracted using the Roche High Pure RNA Isolation Kit (Roche) according to the manufacturer’s instructions. The purified RNA was then quantified using a Nanodrop 2000 UV–Vis Spectrophotometer (Thermo Scientific). A High Capacity Reverse Transcriptase Kit (Applied Biosystems) was used to convert RNA to cDNA according to the manufacturer’s instructions. 25-100 ng of total RNA was converted to cDNA depending on the yield of RNA in each experiment.

TaqMan™ Gene Expression Assays (Applied Biosystems) were used to assay the gene expression of *ZEB1, VIM, CDH1* (E-cadherin) and *CDH2* (N-cadherin). *CDKNIB* was used as the endogenous control. TaqMan™ Gene Expression Mastermix was used for all assays according to the manufacturer’s instructions. A TaqMan™ PreAmp Master Mix Kit was used on the *ZEB1* siRNA knockdown cDNA samples to detect *CDH1, CDH2* and for consistency *CDKNIB*. QPCR was performed using the Roche Light Cycler 96 RT-PCR and analysed using Roche LightCycler 96 software. Relative gene expression was determined using the comparative CT method (2^−ΔΔ*CT*^) [57].

### Western blots

Cells (1.25 × 10^6^ cells/10 cm dish) were plated in 10 mL media and allowed to attach overnight. The next day either 2 mL of fresh media or media containing cisplatin was added to give a final concentration of 200 ng/mL. The plates were then incubated for 72 h. The cells were then trypsinised, washed in 10 mL PBS, centrifuged and the supernatant removed. The cell pellets were stored at − 20 °C prior to analysis. Western blots were performed as previously described [5]. Densitometry on a minimum of *n* = 3 biological replicates was performed using Quantity One software (Biorad), using local-background correction. Abundance of protein in arbitrary units was normalised to β-actin for each sample and then each biological series was normalised to IGROV-1 control, IGROV-1 scramble or IGROVCDDP scramble depending on the experiment. Antibodies used for Western blots are described in Table 5.

**Table 5:** Antibodies for Western blotting

Protein	kDa	Host	Supplier	Catalogue	Dilution
β-Actin	42	Mouse	Sigma	A5441	1:10,000
E-cadherin (C-terminal)	120	Mouse	BD Biosciences	610181	1:10,000
N-cadherin	130	Mouse	BD Biosciences	610920	1:2500
Vimentin	57	Mouse	BD Pharmingen	550513	1:3000
ZEB1	200	Rabbit	Cell Signalling Technology	D80D3	1:1000
Anti-mouse HRP	N/A	Sheep	Sigma	A6782	1:1000
Anti-rabbit HRP		Goat	Sigma	A4914	1:1000
Anti-mouse AP		Rabbit	Sigma	A4312	1:1000

### Morphology analysis

Images of IGROV-1, IGROVCDDP and *ZEB1* siRNA treated IGROVCDDP cells were analysed using Image J [15]. Phase contrast images were captured at 100× magnification and saved as a tif file. A minimum of 50 cells per image were manually outlined and then analysed for cell size and circularity. Biological triplicate experiments were analysed for the *ZEB1* siRNA knockdown.

### Statistical analysis of cell line data

All experiments in cell lines were performed at minimum in biological triplicate, and statistical analysis was carried out using Minitab. A two-sample *t* test was used to determine statistical significance with a *p* value cut-off of < 0.05.

### Meta-survival analysis of publicly-available ovarian-cancer datasets

Meta-survival analysis of publicly-available ovarian-cancer datasets was performed as described by [58]. Briefly, gene-expression data sets were downloaded from the Gene Expression Omnibus (http://www.ncbi.nlm.nih.gov/geo/) in the form of raw data files. Ovarian cancer datasets with survival information and at least 50 patients were included. In total, 2051 samples across 12 datasets incorporating 6 different array platforms were used [32–39, 59]. This resulted in gene-expression data for a total of 20,017 Entrez gene IDs across 2051 samples. We combined detailed clinical data with this gene expression data for each of the 12 datasets. Overall survival or progression-free survival from the clinical data were chosen as the survival endpoints. Median expression was used to dichotomise the data, allowing stratification into high and low groups within each of the 12 individual datasets. Once a sample was assigned to a particular group, the 12 datasets were combined and global pooled survival analyses were performed. Survival curves are based on Kaplan–Meier estimates and the log-rank *p* value is shown for difference in survival. Cox regression analysis was used to calculate hazard ratios. The R package survival was used to calculate and plot the Kaplan–Meier survival curves.

## Abbreviations

EMT: Epithelial to mesenchymal transition
RPMI: Roswell Park Memorial Institute
HR: Hazard ratio
OS: Overall survival
PBS: Phosphate buffered saline
